# Supplementary material for: Multiple unfolded protein response pathways cooperate to link cytosolic dsDNA release to stimulator of interferon gene activation
Source: Front Immunol. 2024 Jul 19;15:1358462. doi: 10.3389/fimmu.2024.1358462 (PMC11294172; doi:10.3389/fimmu.2024.1358462)
Supplement: Supplementary file 1 [file DataSheet_1.docx]

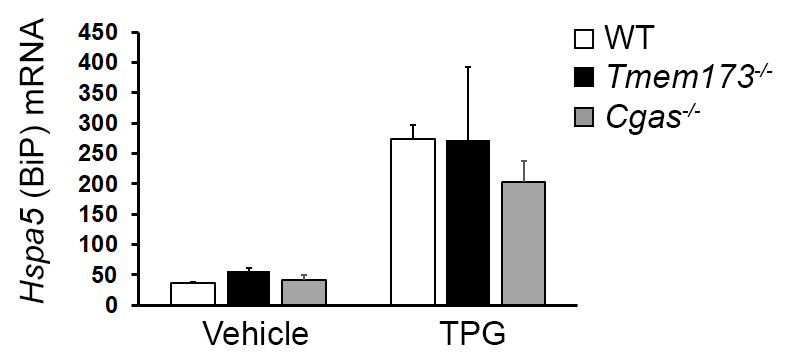


**Figure S1: Thapsigargin induces *Hspa5* (BiP) expression in STING and cGAS knockout macrophages.** Wild type (WT), *Tmem173*-/- or *Cgas*-/- immortalized macrophages were treated with thapsigargin (TPG) for 3h and then harvested for RNA. *Hspa5* expression was detected using qPCR with normalization to 18S rRNA. Bars are from 2 independent experiments with SEM error bars.
