# Supplementary material for: Multiple unfolded protein response pathways cooperate to link cytosolic dsDNA release to stimulator of interferon gene activation
Source: Front Immunol. 2024 Jul 19;15:1358462. doi: 10.3389/fimmu.2024.1358462 (PMC11294172; doi:10.3389/fimmu.2024.1358462)
Supplement: Supplementary file 2 [file DataSheet_2.docx]

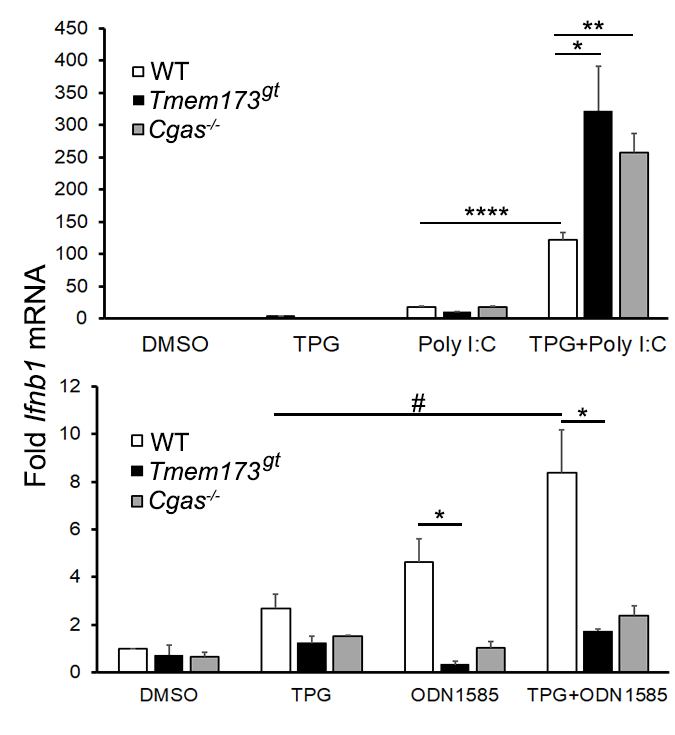


**Figure S2: cGAS and STING play different roles in UPR-TLR induced IFN-β expression for Poly I:C (TLR3) vs ODN-1585 (TLR9)**. WT (wild type, white bars), STING null mutant (Golden Ticket, *Tmem173^gt^* , black bars) or *Cgas*^-/-^ macrophages (gray bars) were stimulated with DMSO vehicle control or 1μM TPG for 1h and then 100 μg/mL poly I:C (top graph) or 3 μM ODN-1585 (bottom graph) for 6h prior to harvest for RNA. mRNA levels were normalized to 18S RNA and WT vehicle stimulated control (fold change). Bars represent means of 2-4 independent experiments (top) or N=2 (bottom) and errors are SEM. #p=0.05, *p<0.05, **p<0.01, ****p<0.001.
