# Supplementary material for: Multiple unfolded protein response pathways cooperate to link cytosolic dsDNA release to stimulator of interferon gene activation
Source: Front Immunol. 2024 Jul 19;15:1358462. doi: 10.3389/fimmu.2024.1358462 (PMC11294172; doi:10.3389/fimmu.2024.1358462)
Supplement: Supplementary file 3 [file DataSheet_3.docx]

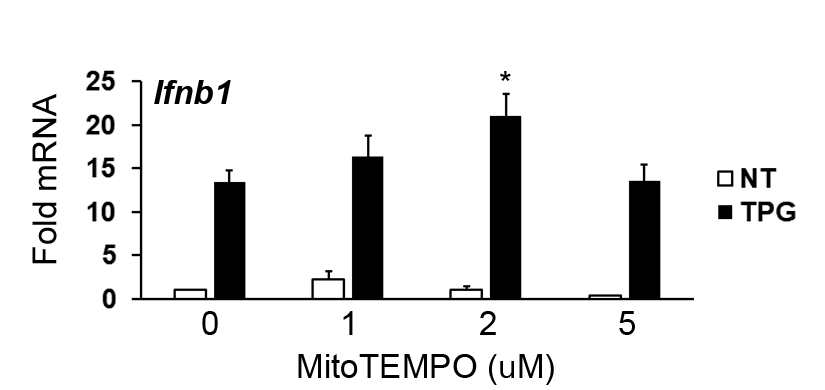


**Figure S3: MitoTEMPO does not decrease Thapsigargin-induced IFN-β expression.** Immortalized macrophages were treated 1h with varying concentrations of MitoTEMPO, the mitochondrial superoxide scavenger, followed by 3h 1 μM Thapsigargin. RNA levels (Fold RNA) were quantitated using qPCR with normalization to 18S rRNA and DMSO vehicle treated (NT) control (set=1). Bars represent means and SEM of 2-6 independent experiments. *p<0.05 in comparison with TPG treated control in the absence of MitoTEMPO.
