# Supplementary material for: Multiple unfolded protein response pathways cooperate to link cytosolic dsDNA release to stimulator of interferon gene activation
Source: Front Immunol. 2024 Jul 19;15:1358462. doi: 10.3389/fimmu.2024.1358462 (PMC11294172; doi:10.3389/fimmu.2024.1358462)
Supplement: Supplementary file 4 [file DataSheet_4.docx]

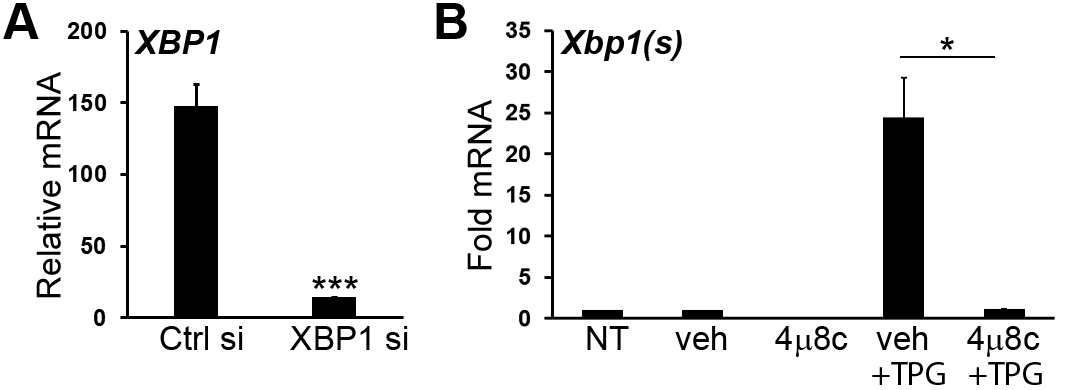


**Figure S4: XBP1 knockdown and 4μ8c inhibition of XBP1 mRNA splicing**. A) HeLa cells were transfected with control (Ctrl) or XBP1 siRNA. After 24h, mRNA was quantitated by qPCR with normalization to 18S rRNA. Bars represent means and SEM of 3 independent experiments. XB1 siRNA knocks down mRNA expression by >90%, ***p=0.0023 vs control siRNA. B) iMac cells were not treated (NT), pre-treated with DMSO (veh) or 10 μM 4μ8c followed by 1 μM TPG for 3h. *p<0.05. Spliced *Xbp1* mRNA was detected by qPCR with normalization to 18S and to NT control (set=1). Results are from 1 experiment in duplicate (SD error bars) and representative of N=3. Similar results were obtained in HeLa and A549 cells.
