# Supplementary material for: Multiple unfolded protein response pathways cooperate to link cytosolic dsDNA release to stimulator of interferon gene activation
Source: Front Immunol. 2024 Jul 19;15:1358462. doi: 10.3389/fimmu.2024.1358462 (PMC11294172; doi:10.3389/fimmu.2024.1358462)
Supplement: Supplementary file 5 [file DataSheet_5.docx]

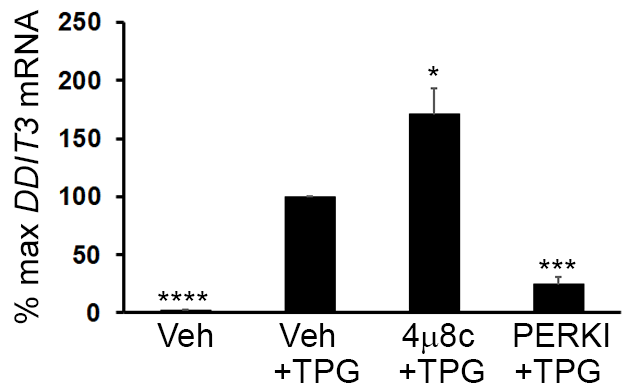


**Figure S5: The PERK inhibitor GSK2606414, but not 4μ8c, blocks TPG-dependent CHOP (*DDIT3*) upregulation**. HeLa cells were pretreated with DMSO vehicle (Veh), the IRE1 inhibitor 4μ8c, or the PERK inhibitor (GSK2606414, PERKI) for 30 minutes, followed by thapsigargin (TPG) for 3 hours. RNA was harvested and cDNA quantitated by qPCR by normalization to 18S rRNA and then to vehicle + TPG (set=100%). Results are from 3 independent experiments performed in duplicate. P-values are vs. vehicle + TPG, * p<0.05, ***p<0.005, ****p<0.001.
