# Supplementary material for: Multiple unfolded protein response pathways cooperate to link cytosolic dsDNA release to stimulator of interferon gene activation
Source: Front Immunol. 2024 Jul 19;15:1358462. doi: 10.3389/fimmu.2024.1358462 (PMC11294172; doi:10.3389/fimmu.2024.1358462)
Supplement: Supplementary file 6 [file DataSheet_6.docx]

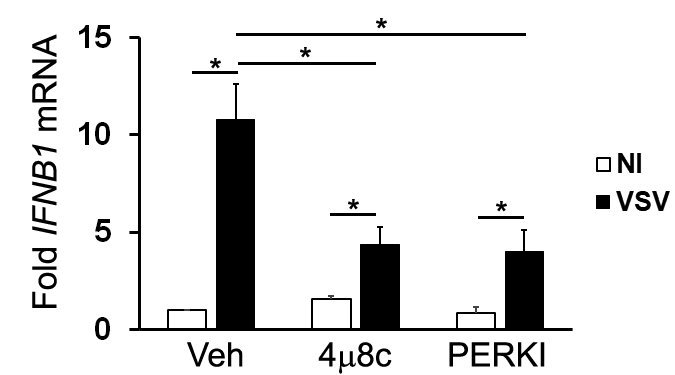


**Figure S6: IRE1 and PERK inhibitors decrease VSV-induced IFN-β expression in HeLa cells**. HeLa cells were pre-treated with DMSO vehicle control or the IRE1 inhibitor 4μ8c or a PERK inhibitor (PERKI) for 30 minutes followed by 6 hours VSV infection. IFN-β mRNA was quantitated by qPCR with normalization to 18SrRNA and to vehicle treated uninfected (NI) control. Results are from 3 independent experiments with SEM, *p<0.05 in pairwise comparisons.
