# Supplementary material for: Multiple unfolded protein response pathways cooperate to link cytosolic dsDNA release to stimulator of interferon gene activation
Source: Front Immunol. 2024 Jul 19;15:1358462. doi: 10.3389/fimmu.2024.1358462 (PMC11294172; doi:10.3389/fimmu.2024.1358462)
Supplement: Supplementary file 7 [file DataSheet_7.docx]

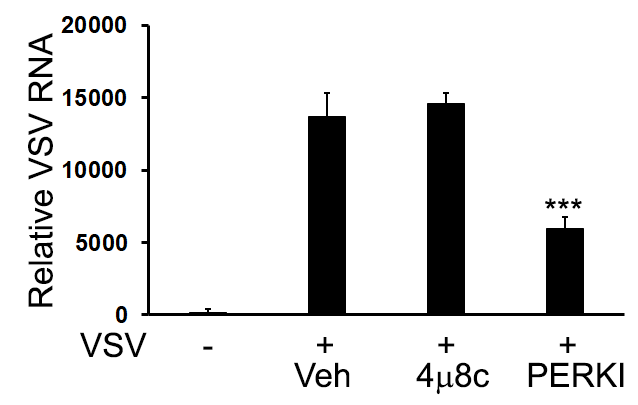


**Figure S7: Effect of UPR inhibitors on VSV replication**. A549 cells were pre-treated for 30 minutes with DMSO vehicle, 4μ8c, or PERK inhibitor (PERKI) and then infected with an MOI=1 of VSV for 24h. VSV replication was detected using qPCR for genomic VSV with normalization to host 18S rRNA. Results are from an experiment in triplicate with SD, and representative of 2 independent experiments. ***p<0.005 vs. vehicle+VSV.
